# Shipping amphorae and shipping sheep? Livestock mobility in the north-east Iberian peninsula during the Iron Age based on strontium isotopic analyses of sheep and goat tooth enamel

**DOI:** 10.1371/journal.pone.0205283

**Published:** 2018-10-31

**Authors:** Silvia Valenzuela-Lamas, Hector A. Orengo, Delphine Bosch, Maura Pellegrini, Paul Halstead, Ariadna Nieto-Espinet, Angela Trentacoste, Sergio Jiménez-Manchón, Dani López-Reyes, Rafel Jornet-Niella

**Affiliations:** 1 Consejo Superior de Investigaciones Científicas- Institució Milà i Fontanals (CSIC-IMF), Barcelona, Spain; 2 McDonald Institute for Archaeological Research, University of Cambridge, Cambridge, United Kingdom; 3 Laboratoire Géosciences, CNRS- Université Montpellier, UMR-5243, Montpellier, France; 4 School of Archaeology, University of Oxford, Oxford, United Kingdom; 5 Department of Archaeology, University of Sheffield, Sheffield, United Kingdom; 6 Archéologie des Sociétés Méditerranéennes, UMR 5140, Labex ARCHIMEDE program IA- ANR-11-LABX-0032-01, Univ Paul-Valéry, CNRS, MCC, Montpellier, France; 7 Arqueovitis sccl, Avinyonet del Penedès, Spain; 8 Àrea de Prehistòria i Arqueologia, Universitat de Barcelona, Barcelona, Spain; New York State Museum, UNITED STATES

## Abstract

Animal mobility is a common strategy to overcome scarcity of food and the related over-grazing of pastures. It is also essential to reduce the inbreeding rate of animal populations, which is known to have a negative impact on fertility and productivity. The present paper shows the geographic range of sheep provisioning in different phases of occupation at the Iron Age site of Turó de la Font de la Canya (7^th^ to 3^rd^ centuries BC). Strontium isotope ratios from 34 archaeological sheep and goat enamel, two archaeological bones and 14 modern tree leaves are presented. The isotopic results suggest that sheep and goats consumed at the site were reared locally (within a few kilometres radius) during the whole period of occupation. The paper discusses the isotopic results in light of the socio-political structure of this period, as complex, strongly territorial societies developed during the Iron Age in the north-east Iberian Peninsula.

## Introduction

The Bronze and the Iron Ages in Europe witnessed increased social differentiation and territoriality. This is reflected in the archaeological record in changing settlement pattern (from open-air sites on the plains to fortified sites on hills), the expansion and progressive complexity of fortifications, and the spread of warrior equipment and weapons in some tombs. These processes thus apparently involved a significant increase in warfare and, probably, in the maintenance of boundaries between territories [1–8]. In the north-east of the Iberian peninsula (present-day Catalonia), the spread of iron technology during the 8^th^-7^th^ centuries BC coincided with the first evidence of rectangular stone buildings, urbanism and fortifications [4, 7, 9]. Iron Age animal husbandry, which did not change dramatically compared to the Late Bronze Age [10–11], was characterised by a remarkable predominance of sheep and goats and by the small size of domestic cattle, sheep/goats and pigs [10–16]. This changed only slightly in the 3^rd^ century BC, when increased consumption of pigs and a slight increase in animal size are attested [10–14, 16], coinciding in time with the expansion of urban centres in the area [9, 17–19].

**Fig 1:**
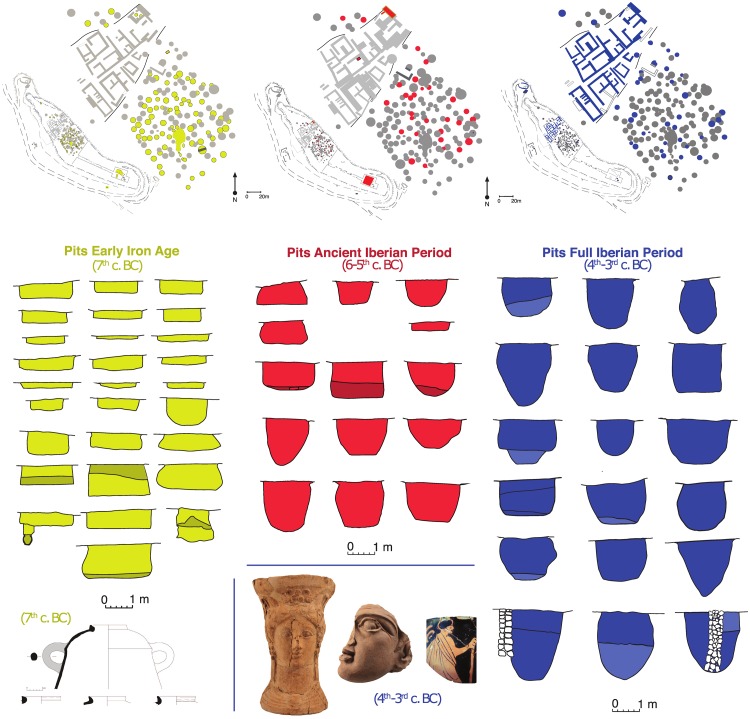
Plan of the site, sections of some silos, and examples of Mediterranean imports dated from the early Iron Age (7^th^ century BC) and the Full Iberian Period (4^th^ - 3^rd^ centuries BC).

**Fig 2:**
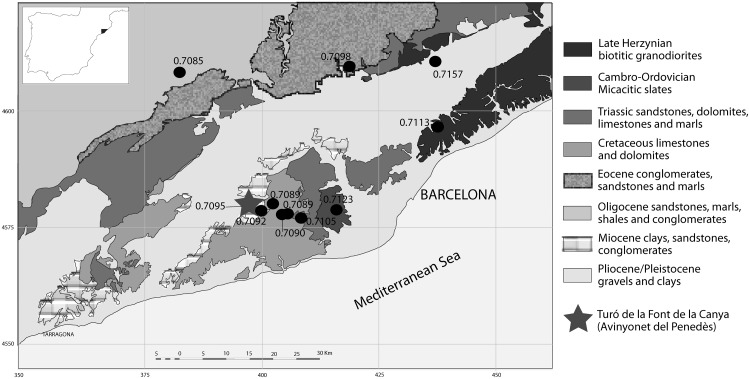
Location of the site (star) and the sampling locations of modern tree leaves and archaeological bones used to assess the bioavailable strontium of the different geological formations surrounding the site. The site is on a Miocene plateau surrounded by Pliocene and Pleistocene sediments.

The site named Turó de la Font de la Canya (Barcelona, Spain) was an important point for cereal storage and trade with other cultures of the Mediterranean, as suggested by the presence of numerous subterranean ‘silos’ suitable for cereal storage, together with imports of Phoenician, Greek and other origins (Fig 1; [20–22]). The site is located on a small promontory (230 m a.s.l.), about 15 km from the present-day coastline and about 40 km south-west of Barcelona [22] (Fig 2). Its occupation spanned the 7^th^ to 2^nd^ centuries BC, that is, from the spread of iron metallurgy in the area to the period of the Roman conquest.

The analysis of strontium isotope ratios from tooth enamel is now a well-established method for exploring human and animal mobility [23–32]. The aim of this study is to characterise the geographical range of sheep provisioning for this important site through its period of occupation, and thus to assess its degree of connectivity regarding meat provisioning through the Iron Age. In other words, we wanted to know whether sheep were moving as much as other materials, such as pottery and other goods recovered from the site [22]. A previous study on seven sheep teeth from this site suggested that sheep were reared locally [33]. The present work significantly expands the number of sheep teeth analysed and covers different phases of occupation, allowing us to look for variations in the geographical range of meat provisioning through time; in this study, we also analysed a few goat teeth. In order to determine the baseline of strontium isotope variability, archaeological bones and modern leaves collected from trees growing on different geological units around the site were also analysed as a basis for comparison with archaeological results.

### Strontium isotope analysis

Strontium substitutes for calcium and occurs as a trace element in biogenic tissues, including the hydroxyapatite of teeth and bones [23, 34–35]. The Sr isotopic ratio (^87^Sr/^86^Sr) varies in different geological formations according to the age and original rubidium (Rb) /strontium (Sr) ratio of the bedrock, leading to high radiogenic ^87^Sr/^86^Sr ratios in old or crustal rocks, and low ^87^Sr/^86^Sr ratios in young or mantle rocks [23, 36]. The ^87^Sr/^86^Sr isotope composition of plants reflects the strontium isotopic ratios of the underlying bedrock, as biologic processes involved during plant growth do not entail isotopic fractionation of strontium isotopes [37–38]. Other factors affecting strontium ratios in plants include a significant contribution of rainfall water [39] atmospheric pollution and the use of modern fertilizers [40–44].

In the case of skeletal material, the ^87^Sr/^86^Sr isotope composition derives from the food and drink ingested by the animal [45–46]. The porosity of bones makes their strontium signature susceptible to diagenetic alteration, but the isotopic signature of tooth enamel bioapatite reflects the period of tooth formation with little subsequent change [47–48]. Therefore, strontium isotope ratios from tooth enamel indicate the type of geological formation from which food and water were sourced during the period of mineralization of the tooth analysed [23–38, 49].

### Geology at the site

The site of Turó de la Font de la Canya is located on a coastal promontory on the south-west margin of the Catalan Coastal Range. From a geological perspective, this mountain range is characterised by fragmented outcrops of mainly Mesozoic and Tertiary sedimentary formations overlying infra-Silurian and Varingian batholith and other Palaeozoic metamorphic rocks [50]. The settlement is located on Miocene clays, sandstones and conglomerates and is surrounded by Plio-Pleistocene alluvia to the west and north, and by Cretaceous limestones to the east and south (Fig 2). In the vicinity of the site, within 10–15 km, other sedimentary (Triassic sandstones, dolomites, limestones and marls) and metamorphic (Cambro-Ordovician Micacitic slates) formations also outcrop. Further to the north-east, an extensive outcrop of Late Hercynian biotitic granodiorite is also present.

## Materials and methods

The analysed archaeological material totals 30 sheep and four goat teeth (including seven sheep teeth previously reported [33]) from different levels of occupation and silos filled with domestic debris. Sheep and goat identification followed usual criteria [51–53]. The selected teeth correspond to second and third molars dated from different phases of occupation of the site: nine teeth dated from the early Iron Age (7^th^ century BC), nine from the Middle Iron Age (6^th^ -5^th^ centuries BC), and 16 from the Late Iron Age (4^th^ -3^rd^ centuries BC). Despite the higher inter-individual variation in the enamel mineralization of the third molar in comparison to the second [54–55], as well as the possible averaging of isotope ratios [56], third molars were selected because they were easily identifiable even when isolated. In all cases, only fully erupted teeth (i.e. in wear) from different individuals were chosen for analysis.

The enamel samples were prepared for strontium isotope analysis following standard practices described in previous studies [24, 33, 57]. The tooth enamel surface was first mechanically abraded to remove all dentine and cementum to a depth of 100 μm using a tungsten carbide dental burr. A transversal slice of enamel about 2mm wide was cut above the enamel root junction (ERJ) from the protoconid of each tooth using a diamond cutter disc coupled to a dentist’s drill. On ten teeth, two more transversal slices of enamel about 2mm wide were cut at the middle and top of the tooth crown (about 9mm and 15mm from the enamel root junction) to analyse potential strontium variations during the period of enamel mineralisation. Although some variation may occur between breeds, mineralisation of sheep third molars starts about 9–12 months, and finishes about 22–34 months [58–59]. Consequently, sequential sampling allows us to observe whether animals moved between different geological layers during enamel mineralisation [49, 56, 60–61]. These ten teeth were added to the seven teeth published in our previous study [33] thus totalling 17 teeth sequentially sampled (minimum 5 teeth per chronological period). The resulting samples were transferred to a clean (class 100, laminar flow hood) working area in the Laboratoire de Géosciences (Montpellier, France) for further preparation. This involved ultrasonic cleaning to remove adhering material and immersion in 60°C water for an hour for further cleaning. After each cleaning phase the sample was rinsed three times on MilliQ high purity de-ionized water. A final cleaning step, in 5% acetic acid for half an hour in an ultrasonic bath, followed by rinsing three or four times with pure water, was also performed in order to be sure that potential remaining diagenetic effects were removed. Once cleaned and dried in a laminar flow hood, the samples were weighed in pre-cleaned Teflon beakers. The samples were then dissolved in Teflon distilled nitric acid (8 M HNO_3_). Strontium was collected using standard resin columns (Eichrom Sr-Spec resin) and then loaded onto single rhenium filaments with a TaCl_5_ activator. Total chemistry blanks were less than 20pg and thus negligible for this study. The strontium isotope composition was determined with a solid-source thermal-ionization mass spectrometer at the Labogis of Nîmes University (Thermo Finnigan TRITON TI). Repeated measurements of the international standard for ^87^Sr/^86^Sr NBS 987 gave a mean value of 0.710251± 0.000018 for static analysis (2 s, n = 9).

In order to assess the variability of bio-available strontium ratios in the vicinity of the site and surrounding geological areas, 14 modern leaves from oak, pine and strawberry trees growing on different geological formations were also analysed (see Table 1 for details, specimen name and precise location). Six of them originated from forests protected as a natural reserve (Natural Park of Garraf, Sant Llorenç del Munt i l’Obac, Montseny, and Montnegre-Corredor), and eight from trees growing on non-cultivated fields far (>100m) from human activities and rivers or streams. The field permits were granted by Diputació de Barcelona. Each sampling location was recorded using a hand-held GPS device. The preparation protocol was adapted from the one described in [40]. Samples of modern leaves were crushed in a Retsch 200ZM grinder, and then weighed in pre-cleaned pressure vessels in a clean laboratory environment. They were dissolved in Teflon distilled nitric acid (8 M HNO_3_) overnight at room temperature. Further acid and a trace of H_2_O_2_ were added, before the samples were processed in a microwave oven at 175°C for 20 minutes. The samples obtained were then dried overnight on a hotplate prior to a secondary oxidation stage which repeated the whole process. The samples were converted to chloride in a solution of 6 M HCl, then dried and taken up again in 2N HNO_3_ prior to strontium separation using standard resin columns (Eichrom Sr-Spec resin).

**Table 1:** Strontium isotopic ratios (^87^Sr/^86^Sr) obtained on modern tree leaves and sheep bones from different geologic formations. Coordinates ETRS89 UTM31N.

Sample	W	N	Era	Period	Epoch	Bedrock	Species	^87^Sr/^86^Sr	Error (2s)	Geological layer
VI-11	329415.2	4603851.5	Cenozoic	Quaternary	Pleistocene	Gravels with lutite matrix and sandy banks	Sheep bone	0.708589	±0.000004	Qvpu
GAR-5	401093.85	4578084.81	Cenozoic	Neogene	Late Miocene	Calcarenites	Evergreen oak	0.709226	±0.000007	NMe
TFC 50	397472	4580693	Cenozoic	Neogene	Middle Miocene	Clays, sandstones and conglomerates	Evergreen oak	0.709508		Nmag
TFC 51	397472	4580693	Cenozoic	Neogene	Middle Miocene	Clays, sandstones and conglomerates	Oak	0.709528		Nmag
VG-020	449065.3	4616603.9	Cenozoic	Neogene	Miocene	Lenticular levels of conglomerates with arcsic sandy matrix	Evergreen oak	0.715757	±0.000003	NMcga
VG-002	331435.8	4635310.3	Cenozoic	Paleogene	Oligocene	Conglometrates	Oak	0.709304	±0.000005	Pogm1
VI-98	291167.6	4611828.6	Cenozoic	Paleogene	Oligocene	Shales and sandstones	Sheep bone	0.709606	±0.000006	POmgc4
VG-001	324446.7	4630001.3	Cenozoic	Paleogene	Eocene-Oligocene	Gray marls	Pine	0.708506	±0.000007	PEOx
VG-025	380229.6	4606831.3	Cenozoic	Paleogene	Eocene-Oligocene	Marls, limestones and sandstones	Pine	0.708541	±0.000005	PEOmg
VG-022	421143.4	4610785.2	Cenozoic	Paleogene	Eocene	Heterometric conglomerates	Evergreen oak	0.709820	±0.000003	PEcg
GAR-6	401454.53	4578621.58	Mesozoic	Cretaceous	Late Cretaceous	Calcareous and dolomitic	Pine	0.708977	±0.000012	CVBcd
GAR-3	405910.83	4576288.54	Mesozoic	Jurassic- Cretaceous	Upper Jurassic- Lower Cretaceous	Calcareous and dolomitic	Evergreen oak	0.709063	±0.000008	Jd
GAR-4	405910.83	4576288,53	Mesozoic	Jurassic- Cretaceous	Upper Jurassic- Lower Cretaceous	Calcareous and dolomitic	Evergreen oak	0.708923	±0.000007	Jd
GAR-1	409109.00	4575181.04	Mesozoic	Triassic	Middle—Late Triassic	Calcareous and dolomitic	Evergreen oak	0.710552	±0.000092	Tm2
VG-024	443688.4	4602000.2	Paleozoic	Carboniferous-Permian	Carboniferous-Permian	Granodiorites and alcaline granites	Strawberry tree	0.711321	±0.000005	Ggd
GAR-7	416507.47	4579214.7	Paleozoic	Cambro-Ordovician	Cambro-Ordovician	Micacitic slates	Evergreen oak	0.712337	±0.000006	ÇOrp

Despite every effort was made to collect reliable samples from the Pleistocene sediments of the Vallès-Penedès valley, it proved difficult due to the high degree of anthropic impact on the landscape–buildings, agriculture–. We therefore took two bone samples from two archaeological sites located on Pliocene and Pleistocene sediments further away to the West (Table 1) as bone tissue absorbs the strontium signature of the burying environment [47–48]. The bone sample preparation followed the same protocol described above for enamel samples.

## Results

### Modern samples

**Fig 3:**
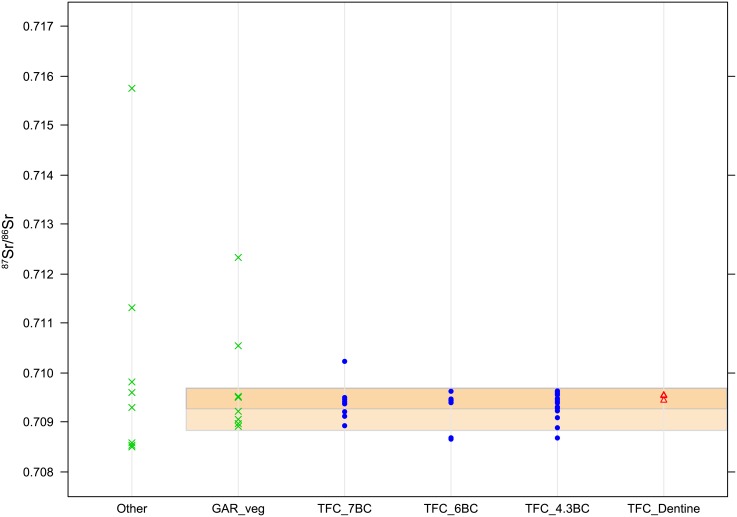
Strontium isotopic ratios (^87^Sr/^86^Sr) obtained from 14 modern tree leaves and two archaeological bones (green crosses), 34 archaeological sheep enamel from Turó de la Font de la Canya (blue dots) and three archaeological sheep dentine from Turó de la Font de la Canya (red triangles). The dark orange band indicates the strontium isotopic range of the Miocene bedrock where the site is located, and the light orange band indicates the range of the Jurassic and Cretaceous sediments neighbour to the site. ‘GAR’ vegetal samples refer to the samples collected on the Garraf mountain area (sample codes starting with GAR on Table 1), ‘Other’ refer to all the other areas (see Table 1 for sample details).

Table 1 and Fig 3 (left) show the strontium ^87^Sr/^86^Sr isotopic ratios obtained from the 14 modern leaf samples and two archaeological bones sourced from different geological formations neighbouring the site. The results indicate that the strontium signature of the site is around 0.7095, and that the potential strontium variation in the surroundings of the site ranges between 0.7089 and 0.7123. The neighbour Jurassic and Cretaceous bedrock of the Garraf mountain displays a range between 0.7089 and 0.7090. Further to the north, the Triassic dolomites gave a signal of 0.7105, and further to the north-east, the Palaeozoic granodiorites and micacitic slates of the littoral mountains display strontium ratios between 0.7113 and 0.7123. In the Vallès, the sample collected on Miocene sediments close to Palaeozoic sediments of the Montseny gave a ^87^Sr/^86^Sr value of 0.7157, and the sample from the Eocene conglomerates in the West a ^87^Sr/^86^Sr ratio of 0.7098. The Eocene- Oligocene marls and the Pleistocene sediments of the Catalan Central Depression had values around 0.7085 and, further to the West (130 Km distant from Turó de la Font de la Canya) the Oligocene bedrock displayed values comprised between 0.7093 and 0.7095. All these strontium ratios are consistent with other measurements from similar geological formations in the Iberian Peninsula and elsewhere [29–30, 32, 38].

### Archaeological samples

Table 2 and Fig 3 show the results of the 34 archaeological sheep and goat teeth from Turó de la Font de la Canya. The vast majority of strontium ^87^Sr/^86^Sr isotopic ratios (n = 27), including those for all four goats, range between 0.7091 and 0.7096, compatible with the values attested on the Miocene sediments where the site is located. Six teeth (one of 7^th^ century BC, two of 6^th^ -5^th^ centuries BC, and three of 4^th^ -3^rd^ centuries BC) have values in the range 0.7086–0.7090, which could correspond to Cretaceous sediments from the neighbouring Garraf mountains (*circa* 4km away). Only one tooth dated to the 7^th^ century BC has a value of 0.7102 (sample 37–1, SU 1549), which could be compatible with the Triassic limestone and dolomites present further away in the Garraf (*circa* 10km from the site).

**Table 2:** Strontium isotopic ratios (^87^Sr/^86^Sr) obtained from archaeological sheep and goats enamel. **Sample located at the base of each tooth, about 1mm above the enamel root junction**. In the table, ‘s/g’ refers to specimens identified as ‘sheep/goat’.

Sample code	SU	Tooth	Species	Chrono	Wear stage (Payne 1987)	^87^Sr/^86^Sr	Error (±2sigma)	Reference
25–1	SU 1233	m2 inf	sheep	7th BC	9A	0.709378	0.000004	This study
30–1	SU 1549	m3 inf	goat	7th BC	10G	0.709418	0.000004	This study
32–1	SU 1549	m3 inf	sheep	7th BC	11G	0.709218	0.000004	This study
33–1	SU 1549	m2 inf	sheep	7th BC	9A	0.708935	0.000003	This study
34–1	SU 1549	m3 inf	goat	7th BC	11G	0.709379	0.000004	This study
37–1	SU 1549	m3 inf	sheep	7th BC	11G	0.710236	0.000005	This study
38–1	SU 1549	m3 inf	sheep	7th BC	5A	0.709126	0.000008	This study
41–1	SU 1649	m2 inf	sheep	7th BC	5A	0.709505	0.000004	This study
59–1	SU 1670	m2 inf	sheep	7th BC	10A	0.709464	0.000004	This study
137–1	SU 1710	m2 inf	sheep	6th BC	9A	0.709631	0.000004	This study
139–1	SU 1710	m3 inf	sheep	6th BC	6G	0.709402	0.000004	This study
47–1	SU 1616	m3 inf	sheep	6th BC	11G	0.709452	0.000008	This study
712–1	SU 1710	m3 inf	sheep	6th BC	9G	0.709441	0.000015	This study
714–1	SU 1710	m3 inf	sheep	6th BC	11G	0.709475	0.000004	This study
716–1	SU 1710	m3 inf	goat	6th BC	6G	0.709401	0.000005	This study
717–1	SU 1710	m3 inf	sheep	6th BC	11G	0.708664	0.000005	This study
718–1	SU 1710	m3 inf	sheep	6th BC	11G	0.708690	0.000003	This study
719–1	SU 1710	m3 inf	goat	6th BC	5G	0.709445	0.000008	This study
53–1	SU 1696	m2 sup	s/g	4th-3rd BC	9A	0.709097	0.000004	This study
54–1	SU 1696	m3 sup	s/g	4th-3rd BC	9A	0.709486	0.000003	This study
13–1	SU 1615	m2 sup	s/g	4th BC	9A	0.709569	0.000005	This study
15–1	SU 1615	m3 sup	s/g	4th BC	4A	0.709309	0.000003	This study
17–1	SU 1646	m3 inf	sheep	4th BC	9G	0.709394	0.000006	This study
58–1	SU 1660	m3 inf	sheep	4th BC	9G	0.708687	0.000004	This study
SU1022	SU 1022	m3 inf	sheep	4th BC	2A	0.709557		Valenzuela-Lamas et al 2016
SU1030	SU 1030	m3 inf	sheep	4th BC	10G	0.709430		Valenzuela-Lamas et al 2016
SU1081	SU 1081	m3 inf	sheep	4th BC	6G	0.709235		Valenzuela-Lamas et al 2016
SU1087A	SU 1087	m3 inf	sheep	4th BC	5G	0.708894		Valenzuela-Lamas et al 2016
SU1087B	SU 1087	m3 inf	sheep	4th BC	6G	0.709471		Valenzuela-Lamas et al 2016
1–1	SU 1090	m2 inf	sheep	3rd BC	7A	0.709622	0.000004	This study
2–1	SU 1090	m2 inf	sheep	3rd BC	4A	0.709287	0.000005	This study
44–1	SU 1769	m2 sup	s/g	3rd BC	5A	0.709638	0.000004	This study
SU1090A	SU 1090	m3 inf	sheep	3rd BC	4A	0.709592		Valenzuela-Lamas et al 2016
SU1090B	SU 1090	m3 inf	sheep	3rd BC	11G	0.709314		Valenzuela-Lamas et al 2016

**Fig 4:**
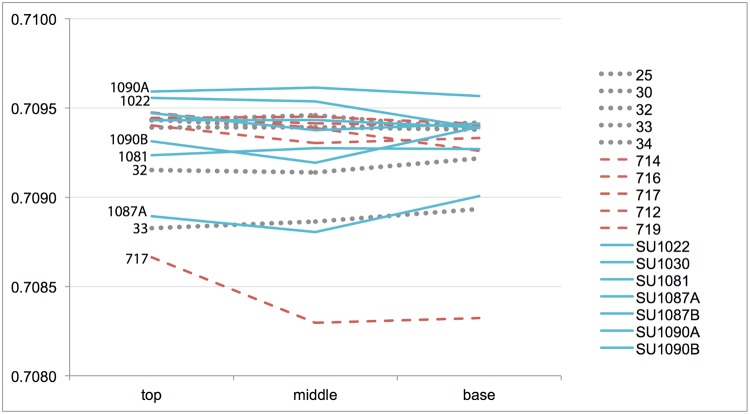
Strontium isotopic ratios (^87^Sr/^86^Sr) of the 17 teeth sequentially sampled (top, middle and bottom of tooth enamel). Grey dots refer to teeth dated from the 7^th^ century BC, red dashed-lines to teeth from 6^th^ century BC and blue lines to teeth dated from the 4^th^ and 3^rd^ centuries BC.

The 17 teeth for which sequential sampling was done display low variability along the tooth crown (Table 3, Fig 4). Again, most teeth have strontium ratios compatible with the local Miocene geology all along the tooth enamel. This indicates that most animals grazed in the vicinity of the site all the year round, and thus suggests that herding was mainly done locally. Only three teeth (717—6^th^ century BC, SU1087A, 4^th^ century BC and SU1090B, 3^rd^ century BC) have notable differences along the tooth height (over 0.000200 between the maximum and the minimum ^87^Sr/^86^Sr value, see Table 3 for details). Two of those animals grazed on an area with strontium values compatible with the Cretaceous sediments from the neighbouring Garraf mountains (teeth 717 and SU1087A), thus reinforcing the idea that some animals arrived to the site from other locations. In this respect, no seasonal pattern of mobility is evidenced between the Garraf mountains and the Plio-Pleistocene valley, but some teeth have similar degrees of variation along the tooth crown. This is the case of teeth 32 and 33 (7^th^ century BC) as well as SU1087A and SU1090B (3^rd^ century BC, see Fig 4). This suggests that some animals may have moved around in a similar way, although this was not the case for most animals.

**Table 3:** Strontium isotopic ratios (^87^Sr/^86^Sr) obtained from 17 archaeological sheep and goats enamel. Samples located at the base, middle and top of enamel. Teeth codes are the same as in Table 2.

Sample code	Top	Middle	Base	Chronology	Max	Min	Difference
25	0.709390	0.709392	0.709378	7th BC	0.709392	0.709378	0.000014
30	0.709434	0.709386	0.709418	7th BC	0.709434	0.709386	0.000048
32	0.709153	0.709139	0.709218	7th BC	0.709218	0.709139	0.000079
33	0.708826	0.708864	0.708935	7th BC	0.708935	0.708826	0.000108
34	0.709430	0.709460	0.709379	7th BC	0.709460	0.709379	0.000081
714	0.709475	0.709387	0.709259	6th BC	0.709475	0.709259	0.000215
716	0.709401	0.709303	0.709331	6th BC	0.709401	0.709303	0.000099
717	0.708664	0.708297	0.708323	6th BC	0.708664	0.708297	0.000367
712	0.709441	0.709414	0.709398	6th BC	0.709441	0.709398	0.000044
719	0.709445	0.709450	0.709410	6th BC	0.709450	0.709410	0.000040
SU1022	0.709557	0.709537	0.709387	4th BC	0.709557	0.709387	0.000170
SU1030	0.709430	0.709432	0.709391	4th BC	0.709432	0.709391	0.000041
SU1081	0.709235	0.709274	0.709269	4th BC	0.709274	0.709235	0.000039
SU1087A	0.708894	0.708804	0.709006	4th BC	0.709006	0.708804	0.000202
SU1087B	0.709471	0.709375	0.709412	4th BC	0.709471	0.709375	0.000096
SU1090A	0.709592	0.709614	0.709566	3rd BC	0.709614	0.709566	0.000048
SU1090B	0.709314	0.709193	0.709394	3rd BC	0.709394	0.709193	0.000201

## Discussion

The ^87^Sr/^86^Sr data of the modern vegetation and archaeological bone reflect the diversity of the geological settings in the Catalan central coast and central depression. The samples originated from the main geological areas around Turó de la Font de la Canya and provide a first overview of the strontium isotopic ranges from the vicinity of the site to 130 Km away to the West. The sampling of the Pleistocene sediments of the Vallès-Penedès valley proved challenging due to the high degree of human impact in this area, which prevented us to collect reliable samples. The strontium results from the large majority of the archaeological samples (27 out of 34 considering the bottom slice, and 53 out of 64 considering all the samples) are compatible with the geology immediately surrounding the archaeological site. This is consistent with sheep and goats being reared locally in the different phases of occupation, most likely on the Miocene sediments and Pleistocene gravels and clays of the Penedès Valley. Nevertheless, the use of other areas of this valley (the Vallès) cannot be excluded, as the Vallès-Penedès forms a corridor of Plio-Pleistocene alluvial sediments surrounded by older geological formations (see text above and Fig 2, also [50]). The presence of various stone pastoral enclosures in the neighbouring Garraf mountains indicates that livestock was present in this coastal mountain area during the first millennium BC [62–65]. Interestingly, the strontium results obtained so far at Turó de la Font de la Canya suggest that this human community did not pasture its sheep and goats on the Garraf or other neighbouring mountains on a regular basis. Only six teeth out of the 34 analysed have a strontium signature compatible with that of the Cretaceous limestones of the Garraf, despite their proximity (*circa* 4 km as the crow flies). Overall, the limited variability of strontium ratios points towards local rearing of caprines rather than long-distance trade of animals.

The homogeneity of the strontium results contrast with the diversity of imports recovered from the site, which include Cruz del Negro pottery from the Straits of Gibraltar, *Tanit* figurines of Punic origin, a clay mask from the central or eastern Mediterranean, and numerous Campanian A ceramics from Italy in the levels dated from the 4^th^ and 3^rd^ centuries BC among others [20–22]. While the possibility cannot be excluded that the sheep and goats analysed originated from other areas with similar geology, the homogeneity of tooth values in a fairly large sample argues against this. It seems, therefore, that sheep and goats were mainly bred locally, and only ‘prestige’ goods (e.g. Phoenician wine, Greek pottery) arrived from distant sources.

Overall, material cultural data suggest that Turó de la Font de la Canya had an active role in the Mediterranean trade. Conversely, the results from strontium isotopic analysis on sheep and goats suggest that animal were reared locally, and so probably on a small-scale basis, and that caprines slaughtered at the site were mainly reared in the Penedès valley and perhaps also the neighbouring Vallès. This local breeding of livestock may reflect (and be a consequence of) the local socio-political context. The significant change in settlement pattern in the Late Bronze and Iron Ages–from open-air sites located on the plains to fortified settlements located on hills–is thought to reflect increased warfare and territoriality [1, 5, 7, 66], while weapons in some tombs and severed heads at several sites suggest the existence of a military elite at this time [18–19]. Perhaps, even if long-distance maritime trade flourished during the Iron Age across the Mediterranean, long-distance *terrestrial* movements of livestock were difficult in the north-east Iberian Peninsula at this time.

## Conclusions

This study has established baseline strontium isotopic ratio (^87^Sr/^86^Sr) values for the vicinity of the Iron Age site of Turó de la Font de la Canya (Barcelona, Spain) and surrounding geological areas, based on analysis of modern leaves from trees growing on different geological substrates around the site and two archaeological bone samples. Modern leaves provided a consistent and fairly narrow range of local strontium ratio values. This new baseline has been central for the interpretation of the strontium isotopic ratios measured in 34 archaeological sheep and goat tooth enamel samples dated from the 7^th^ to 3^rd^ centuries BC, to explore the geographical range of meat provisioning at Iron Age Turó de la Font de la Canya. Seventeen of these samples were sequentially analysed to observe variations along the tooth crown. The investigated archaeological samples indicate limited variability of strontium values throughout the occupation of the site, and 27 of the 34 samples are compatible with the local geology (^87^Sr/^86^Sr range: 0.7091–7096), while seven samples may be derived from bedrocks as close as 5 km from the site (^87^Sr/^86^Sr: 0.7086–0.7090), and one more (^87^Sr/^86^Sr: 0.7102) is compatible with slightly more distant (about 15 km from the site) geological areas. The sequential sampling suggests that most animals were reared locally all the year round, thus supporting the idea of small scale herding rather than long-distance sourcing. Interestingly, some animals display similar patterns of variation along the tooth crown, thus suggesting that some movement of livestock existed. Overall, the archaeological results suggest that sheep and goats slaughtered at the site were mainly reared in the local area, most notably the Miocene clays and Plio-Pleistocene alluvial sediments of the plain. This contrasts with the role of the site as central point for cereal storage (see Fig 2) as well as the diversity of imports recovered at the site, which suggests that only ‘prestige’ goods (e.g. Phoenician wine, Greek pottery)–rather than animals–were brought to this storage-rich site. It further suggests that crop rather than livestock surpluses financed participation in supra-regional exchange.

The low diversity of strontium ratios of sheep and goats at Turó de la Font de la Canya implies herding over a limited geographical range that may partly have been dictated to the difficulty of long-distance terrestrial movements in the Iron Age, as a result of the emergence of the small, strongly defended territories suggested by the settlement pattern, architecture and finds of weapons. Alternatively, the limited geographical scale of herding may primarily reflect the rearing of livestock in only modest numbers. Further zooarchaeological studies, both macroscopic and isotopic, may clarify which of these rival explanations is more plausible.

## References

[1] Johnson, A.V. & Earle, T. 1987. The Evolution of Human Societies. From Foraging Group to Agrarian State, Stanford, Stanford University Press

[2] Py M. 1993 Les Gaulois du Midi, de la fin de l’âge du Bronze à la conquête romaine. Paris: Hachette.

[3] Brun, P. 1995. From chiefdom to state organization in Celtic Europe. In: Arnold & Gibson (Eds.) Celtic chiefdom, Celtic state, The evolution of complex social systems in prehistoric Europe, pp. 13–25

[4] Asensio, D., Belarte, M.C., Sanmartí, J., Santacana, J. 1998. Paisatges ibèrics. Tipus d’assentaments i formes d’ocupació del territori a la costa central de Catalunya durant el període ibèric ple. In: Los Iberos. Príncipes de Occidente. Actas del Congreso Internacional (Barcelona, Fundación la Caixa), 373–85.

[5] Sanmartí, J., Belarte, M.C. 2001. Urbanización y desarrollo de estructuras estatales en la costa de Cataluña (siglos VII-III a.n.e.), Entre celtas e íberos, Madrid, pp. 161–174.

[6] Collis J. 2003 The Celts: Origins, Myths and Inventions, Tempus Publishing.

[7] López-Cachero J. 2007 Society and economy in the Late Bronze Age and the Early Iron Age in the Northeast of the Iberian Peninsula: an approach from the archaeological sources, *Trabajos de Prehistoria* 64: 99–120.

[8] Kristiansen K. 2016 Interpreting Bronze Age trade and migration in: Kiriatzi E., & Knappett C. (Eds.). *Human Mobility and Technological Transfer in the Prehistoric Mediterranean*. Cambridge University Press, 154–80.

[9] Sanmartí J. 2004 From local groups to early states: the development of complexity in protohistoric Catalonia, *Pyrenae*, 35 (1): 7–42.

[10] Franquesa D., Oltra J., Piña A., Pons E., Saña M., Verdúm E. 2000 La ramaderia en les societats ibèriques del N-E de la Península Ibèrica: diversificació i especialització. Saguntum Extra 3: 153–161.

[11] Albizuri, S., Nieto, A., Valenzuela-Lamas, S. 2010: Canvis en l’alimentació càrnia a Catalunya entre els segles XII I III aC. In: Mata-Pareño, C., Pérez-Jordà, G. and Vives-Ferrándiz, J.(eds.), De la cuina a la taula (Valencia, Saguntum Extra-9), 161–70.

[12] Valenzuela-Lamas, S. 2008. Alimentació i ramaderia al Penedès durant la protohistòria (segles VII-III aC), Premi d’Arqueologia, Memorial Josep Barberà i Farràs, Societat Catalana d’Arqueologia, Barcelona.

[13] Colominas, L. 2009. La gestió dels animals al nord-est de la península ibérica entre els segles V ane-V dne. Proposta metodològica d’integració de les anàlisis arqueozoològiques als estudis de cronologies històriques. Doctoral Thesis. Universitat Autònoma de Barcelona.

[14] Nieto, A. 2012. Entre el consum i l’afecte. La interacció entre els animals i les comunitats protohistòriques de la plana occidental catalana (segles VII-IV aC). PhD thesis, University of Lleida.

[15] Colominas L., Fernández-Rodríguez C., & Iborra-Eres M. P. 2017 Animal Husbandry and Hunting Practices in Hispania Tarraconensis: An Overview. *European Journal of Archaeology*, 20(3), 510–534.

[16] Valenzuela-Lamas S., Albarella U. 2017 Animal Husbandry across the Western Roman Empire: Changes and Continuities, *European Journal of Archaeology* 20 (3): 402–415.

[17] Asensio, D., Morer, J., Rigo, A., Sanmartí, J. 2001. Les formes d’organització social i econòmica a la Cossetània ibèrica: noves dades sobre l’evolució i tipologia dels assentaments entre els segles VII-II a. C.”, dins Martín, A., Plana, R. (dir.) I Taula Rodona Internacional d’Ullastret: Territori polític i terri- tori rural durant l’edat del ferro a la Mediterrània occidental, Monografies d’Ullastret, 2, 253–271.

[18] Asensio, D., Morer, J., Pou, J., Sanmartí, J., Santacana, J. 2005a. Evidències arqueològiques del procés d’emergència d’èlits aristocràtiques a la ciutadella ibèrica d’Alorda Park (Calafell, Baix Penedès)”, in: XIII Col·loqui Internacional d’Arqueologia de Puigcerdà, Món Ibèric als Països Catalans, Homenatge a Josep Barberà i Farràs, 597–614.

[19] Sanmartí J. 2011 From the archaic states to romanization: a historical and evolutionary perspective on the Iberians, Catalan Historical Review 2: 9–32.

[20] Asensio D., Cela X., Morer J. 2005b El jaciment protohistòric el Turó de la Font de la Canya (Avinyonet del Penedès, Alt Penedès): un nucli d’acumulació d’excedents agrícoles a la Cossetània (segles VII-III aC)”, Fonaments, 12: 177–195.

[21] Sanmartí J., Asensio D., Belarte M. C., Noguera J. 2009 Comerç colonial, comensalitat i canvi social a la protohistòria de Catalunya”, in Diloli, J., Sardà, S., Ideologia, pràctiques rituals i banquet al nord-est de la península Ibèrica durant la protohistòria, Citerior, 5, 219–238.

[22] López, D., Asensio, D., Jornet, R., Morer, J. 2015. La Font de la Canya, guia arqueològica: Un centre de mercaderies a la Cossetània ibèrica i origen de la vinya, LlopArt Impressions, 117pgs.

[23] Bentley R.A. 2006 Strontium Isotopes from the Earth to the Archaeological Skeleton: A Review. Journal of Archaeological Method and Theory, 13: 136–87.

[24] Evans J.A., Tatham S., Chenery S.R., Chenery C.A. 2007 Anglo-Saxon animal husbandry techniques revealed though isotope and chemical variations in cattle teeth, Applied Geochemistry 22: 1994–2005.

[25] Pellegrini M., Donahue R. E., Chenery C., Evans J., Lee-Thorp J.A., Montgomery J., et al 2008 Faunal migration in late-glacial central Italy: implications for human resource exploitation. Rapid Communications in Mass Spectrometry 22: 1714–1726. 10.1002/rcm.3521 18537188

[26] Sykes N., White J., Hayes T., Palmer M. 2006: Tracking animals using strontium isotopes in teeth: the role of fallow deer (*Dama dama*) in Roman Britain, Antiquity 80: 948–959.

[27] Chenery C., Müldner G., Evans J., Eckardt H., Lewis M., 2010 Strontium and stable isotope evidence for diet and mobility in Roman Gloucester, UK, Journal of Archaeological Science 37, 150–163.

[28] Neil S., Evans J., Montgomery J., Scarre C. 2016 Isotopic evidence for residential mobility of farming communities during the transition to agriculture in Britain, Royal Society Open Science 3: 150522 10.1098/rsos.150522 26909177 PMC4736932

[29] Goude G., Castorina F., Herrscher E., Cabut S., & Tafuri M. A. 2012 First strontium isotope evidence of mobility in the Neolithic of Southern France. *European Journal of Archaeology*, 15(3), 421–439.

[30] Knipper C., Meyer C., Jacobi F., Roth C., Fecher M., Stephan E., et al 2014 Social differentiation and land use at an Early Iron Age “princely seat”: bioarchaeological investigations at the Glauberg (Germany). *Journal of archaeological science*, 41, 818–835.

[31] Minniti C., Valenzuela-Lamas S., Evans J., Albarella U. 2014 Widening the market. Strontium isotope analysis on cattle teeth from Owslebury (Hampshire, UK) highlights changes in livestock supply between the Iron Age and the Roman period, Journal of Archaeological Science 42: 305–314.

[32] Villalba-Mouco, V., Sauqué, V., Sarsketa-Gartzia, I., Pastor, M.V., le Roux, P., Vicente, D., et al. 2017. Territorial mobility and subsistence strategies during the Ebro Basin Late Neolithic-Chalcolithic: A multi-isotope approach from San Juan cave (Loarre, Spain), Quaternary International,

[33] Valenzuela-Lamas S., Jiménez-Manchón S., Evans J., López D., Jornet R. Albarella U. 2016 Analysis of seasonal mobility of sheep in Iron Age Catalonia (north-eastern Spain) based on strontium and oxygen isotope analysis from tooth enamel: First results, Journal of Archaeological Science: Reports, 6: 828–836.

[34] Ericson J. E. 1985 Strontium isotope characterization in the study of prehistoric human ecology. Journal of Human Evolution, 14(5), 503–514.

[35] Capo R. C., Stewart B. W., & Chadwick O. A. 1998 Strontium isotopes as tracers of ecosystem processes: theory and methods. Geoderma, 82(1–3), 197–225.

[36] Faure G., & Mensing T. M. 2005 Isotopes: principles and applications. John Wiley & Sons Inc.

[37] Graustein W. C. 1989 ^87^Sr/^86^Sr ratios measure the sources and flow of strontium in terrestrial ecosystems In Stable isotopes in ecological research (pp. 491–512). Springer, New York, NY.

[38] Brönimann D., Knipper C., Pichler S.L., Röder B., Rissanen H., Stopp B., et al 2018 The lay of land: Strontium isotope variability in the dietary catchment of the Late Iron Age proto-urban settlement of Basel-Gasfabrik, Switzerland, Journal of Archaeological Science: Reports, 17, 279–292.

[39] Evans J. A., Montgomery J., Wildman G., & Boulton N. 2010 Spatial variations in biosphere ^87^Sr/^86^Sr in Britain. Journal of the Geological Society, 167(1), 1–4.

[40] Techer I., Lancelot J., Descroix F., Guyot B. 2011 About Sr isotopes in coffee ‘Bourbon Pointu’ of the Réunion Island, Food Chemistry 126: 718–724.

[41] Böhlke J. K., & Horan M. 2000 Strontium isotope geochemistry of groundwaters and streams affected by agriculture, Locust Grove, MD. Applied Geochemistry, 15(5), 599–609.

[42] Maurer A.-F., Galer S.J.G., Knipper C., Beierlein L., Nunn E.V., Peters D., et al 2012 Bioavailable ^87^Sr/^86^Sr in different environmental samples—Effects of anthropogenic contamination and implications for isoscapes in past migration studies, Science of the Total Environment 433: 216–229. 10.1016/j.scitotenv.2012.06.046 22796412

[43] Clauer N., & Semhi K. 2016 An evaluation of soil–plant–water interactions based on the ^87^Sr/^86^Sr, 1/Sr, Ca/Sr, K/Rb and K/Ca ratios of the respective components. Environmental Earth Sciences, 75(8), 690.

[44] Techer I., Medini S., Janin M., & Arregui M. 2017 Impact of agricultural practice on the Sr isotopic composition of food products: Application to discriminate the geographic origin of olives and olive oil. Applied geochemistry, 82, 1–14.

[45] Comar C. L., Russell R. S., & Wasserman R. H. 1957 Strontium-calcium movement from soil to man. Science, 126(3272), 485–492. 13467231 10.1126/science.126.3272.485

[46] Toots H., & Voorhies M. R. 1965 Strontium in fossil bones and the reconstruction of food chains. Science, 149(3686), 854–855. 10.1126/science.149.3686.854 17737382

[47] Price T. D., Burton J. H., & Bentley R. A. 2002 The characterization of biologically available strontium isotope ratios for the study of prehistoric migration. Archaeometry, 44(1), 117–135.

[48] Bentley R.A., Price T.D., Lüning J., Gronenborn D., Wahl J., Fullagar J. 2002 Prehistoric Migration in Europe: Strontium Isotope Analysis of Early Neolithic Skeletons, Current Anthropology, 45 (5): 799–804.

[49] Balasse M., Ambrose S. H., Smith A. B., & Price T. D. 2002 The seasonal mobility model for prehistoric herders in the south-western Cape of South Africa assessed by isotopic analysis of sheep tooth enamel. Journal of Archaeological Science, 29(9), 917–932.

[50] Santanach P., Casas J. M., Gratacós O., Liesa M., Muñoz J. A., Sàbat F. 2011 Variscan and Alpine structure of the hills of Barcelona: geology in an urban area, Journal of Iberian Geology 37 (2): 121–136.

[51] Payne S. 1985 Morphological distinctions between the mandibular teeth of young sheep, Ovis, and goats, Capra. Journal of Archaeological Science 12, 139–147.

[52] Helmer D. 2000 Discrimination des genres Ovis et Capra à l’aide des prémolaires inférieures 3 et 4 et interpretation des âges d’abattage; l’exemple de Dikili Tash (Grèce). Anthropozoologica 31/Ibex: Journal of Mountain Ecology 5, 29–38.

[53] Halstead P., Collins P., Isaakidou V. 2002 Sorting the Sheep from the Goats: Morphological Distinctions between the Mandibles and Mandibular Teeth of Adult Ovis and Capra, Journal of Archaeological Science 29, 545–553.

[54] Blaise E., & Balasse M. 2011 Seasonality and season of birth of modern and late Neolithic sheep from south-eastern France using tooth enamel δ^18^O analysis. Journal of archaeological Science, 38(11), 3085–3093.

[55] Tornero C., Bălăşescu A., Ughetto-Monfrin J., Voinea V., & Balasse M. 2013 Seasonality and season of birth in early Eneolithic sheep from Cheia (Romania): methodological advances and implications for animal economy. Journal of Archaeological Science, 40(11), 4039–4055.

[56] Montgomery J., Evans J. A., & Horstwood M. S. 2010 Evidence for long-term averaging of strontium in bovine enamel using TIMS and LA-MC-ICP-MS strontium isotope intra-molar profiles. Environmental Archaeology, 15(1), 32–42.

[57] Towers J., Montgomery J., Evans J., Jay M., & Parker Pearson M. 2010 An investigation of the origins of cattle and aurochs deposited in the Early Bronze Age barrows at Gayhurst and Irthlingborough. Journal of Archaeological Science, 37(3), 508–515.

[58] Milhaud G, Nezit J (1991) Développement des molaires chez le mouton. Etude morphologique, radiographique et microdurométrique. Recueil de Médecine Vétérinaire, 167:121–127.

[59] Fricke H.C., O’Neil J.R., 1996 Inter- and intra-tooth variation in the oxygen isotope composition of mammalian tooth enamel phosphate: implications for palaeoclimatological and palaeobiological research. Palaeogeography, Palaeoclimatology, Palaeoecology 126, 91–99.

[60] Viner S., Evans J., Albarella U., Pearson M.P., 2010 Cattle mobility in prehistoric Britain: strontium isotope analysis of cattle teeth from Durrington Walls (Wiltshire, Britain), Journal of Archaeological Science 37, 2812–2820

[61] Bogaard A., Henton E., Evans J. A., Twiss K. C., Charles M. P., Vaiglova P. et al 2014 Locating Land Use at Neolithic Çatalhöyük, Turkey: The Implications of ^87^Sr/^86^Sr Signatures in Plants and Sheep Tooth Sequences, Archaeometry 56: 860–877.

[62] Miret M., & Miret J. 1981 Un assentament d’època romana a la Serra de la Font del Coscó (Avinyonet). Tancats medievals per a bestiar al Massís del Garraf. Miscel·lània Penedesenca, 4, 181–194.

[63] Mestres J., Senabre M.R., Socias J., 1994–1996 L’alt penedès a la primera edat del Ferro: consideracions a l’entorn d’un model d’ocupació del territori, Models d’ocupació, transformació i explotació del territori entre el 1600 i el 500 ane a la Catalunya meridi- onal i zones limítrofes de la depressió de l’Ebre, actes de la taula rodona, (Sant Feliu de Codines, 18/19 de novembre de 1994). Gala 3–5, 247–263.

[64] Cebrià A., Esteve X., Mestres J., 2003 Enclosures a la Serra del Garraf des de la Protohistòria a la Baixa Antiguitat In: Guitart J., Palet J.M., Prevosti M. (Eds.), Territoris Antics a la Mediterrània i a la Cossetania Oriental. Generalitat de Catalunya, Barcelona, pp. 313–316.

[65] Ejarque A., Orengo H. 2009 Legacies of change: the shaping of cultural landscapes in a marginal Mediterranean Mountain Range, the Garraf Massif, North-Eastern Spain. Oxford Journal of Archaeology, 28(4), 425–440.

[66] Flannery K., Marcus J., 2012 The Creation of Inequality: How Our Prehistoric Ancestors Set the Stage for Monarchy, Slavery, and Empire. Harvard University Press, Cambridge.

